# Supplementary material for: Leveraging potential of limpid attention transformer with dynamic tokenization for hyperspectral image classification
Source: PLoS One. 2025 Aug 4;20(8):e0328160. doi: 10.1371/journal.pone.0328160 (PMC12321143; doi:10.1371/journal.pone.0328160)
Supplement: Attachment — Submitted filename: Response to Reviewers .docx [file pone.0328160.s002.docx]

Summary of modifications

Dear Sir,

We reciprocate our sincere thanks to you and esteem reviewers for their valuable suggestions and recommendations. We have tried our best to incorporate these suggestions, which are given below:

**Reviewer #1:**

Clarify the meaning of “limpid” in the context of attention—it’s not a standard term in machine learning, so defining it early would help.

Ans: Thank you for your concern. Yes, limpid is not a standard term in machine learning. In the ViT, self-attention is calculated on the patches to provide a global correlation of the spatial features. Several studies reported that their ViT model, with different names, like the Swin transformer, has a square window-based self-attention mechanism. Axial attention-based ViT calculates attention row and column-wise. In the proposed study, the LSANet attention block is named limpid-shaped attention, which is non-square and calculates attention on the tokens. The limpid-shaped attention has broader receptive fields and linear time complexity.

Add a summary table or bullet points comparing the number of parameters and training time across models.

Ans: Thank you for your valuable suggestion. The training time comparison has been demonstrated in the Figure 10 in section 4.5.5.

**4.5.5. Training time analysis**

The trainable parameters and sample presented in the land covers decide the training time the models take. In the proposed study, training time of each model under the same experimental condition is caclullated on PU, IP, SV and Botswana datasets. As shown in Figure 10, it can observe that the 2D-CNN takes less time on all the datasets due to less trainable parameters. Meanwhile, 3D-CNN has a high training time due to large training parameters. Furthermore, Hybrid CNN has 3D and 2D convolution layers with higher train time than 2D-CNN. The ViT-based methods SF and MF have a higher computation time than classical CNN, except for 3D-CNN, due to the computation of attention in the encoder. The SS1DSwin take relatively less training time due to attention calculation through 1D-CNN layers. The proposed LSANet is designed using lightweight 3D-CNN and 2D-CNN, and attention is calculated through parallel row and column-wise. Due to this, training time is less compared to several methods.

Training Time (s)

| Methods | PU | #REF! | IP | #REF! | SV | #REF! | Botswana | #REF! |
| --- | --- | --- | --- | --- | --- | --- | --- | --- |
| 2DCNN | 248.2 | 1 | 265.2 | 1 | 389.6 | 1 | 104.4 | 1 |
| 3DCNN | 690.4 |  | 836 |  | 624.2 |  | 249.2 |  |
| HybridSN | 568.42 |  | 506.2 |  | 618.6 |  | 250.2 |  |
| CSIL | 568 |  | 576.2 |  | 621.6 |  | 248.4 |  |
| SF | 525.6 |  | 448.2 |  | 825.4 |  | 256.6 |  |
| DSGSF | 485.23 |  | 568.2 |  | 856.8 |  | 265 |  |
| MF | 518.2 |  | 617.6 |  | 857.4 |  | 310.2 |  |
| SS1DSwin | 527.6 |  | 527.4 |  | 685.2 |  | 312.6 |  |
| LSANet | 426.31 |  | 487.52 |  | 514 |  | 215.15 |  |

**Figure 10.** Training time comparison on PU, IP, SV, and Botswana datasets

The trainable parameters in millions (M) and flops in Gigaflops (G) of each model based on 30 bands of the datasets are depicted in Table 13. Table 13 shows that the 3DCNN model has the highest and 2DNN has the lowest trainable parameters. At the same time, the 2DCNN and 3DCNN have high flop values. The transformer-based methods CSIL, SpectralFormer, DSGSF, MorphFormer and SS1DSwin have high flop values. At the same time, the SS1DSwin has 26.05 M trainable parameters. Moreover, the proposed LSANet has 2.3M trainable parameters and 1.27 G flops.

**Table 13.** Parameters and flops comparison

| Model | Parameters (M) | Flops (G) |
| --- | --- | --- |
| 2DCNN | 0.40 | 2.69 |
| 3DCNN | 71.17 | 2.57 |
| HybridSN | 5.12 | 0.26 |
| CSIL | 0.70 | 1.84 |
| SpectralFormer | 0.72 | 1.87 |
| DSGSF | 0.82 | 1.01 |
| MorphFormer | 0.64 | 1.29 |
| SS1DSwin | 26.05 | 0.69 |
| Proposed LSANet | 2.34 | 1.27 |

Consider explaining the intuition behind some design choices (e.g., choice of 3D-CNN size, limpid block configuration).

Ans: Thank you for your valuable suggestion. The HSI contains rich spatial and less spectral features. For the efficient segregation of the land covers from the HSI, it is necessary to extract spectral and spatial features. The 3D-CNN is capable to extract spectral features. At the same time, high number of 3D-CNN layers increases the computation costs. In the proposed study, we utilized single 3D-CNN layer for spectral and spatial features. The 3D-CNN is designed using convolution of size 64, kernel size 3x3 followed by BN (batch normalization) and ReLu activation function. A moderate number of filters such as 64 ensures diverse feature from different filters. At the same time, small kernel size 3x3 capture fine grained spatial features from the edge and boundary region. Moreover, batch normalization stabilizes the training and ReLu activation allows LSANet to learn complex pattern of the HSI.

The limpid shape attention block captures rich contextual information. The feature map is split into multiple limpid shape blocks in which each limpid has equal rows and columns to capture the self-attention. In addition, it is GPU friendly in which parallel computation of attention score can be performed, which results less the training time.

**Reviewer #2:**

The reviewer appreciates authors for their research work. The paper meets the quality level of PLOS one and can be accepted for publication after addressing following major revisions. Thank you.

1. Convolutional neural networks (CNNs), limpid size attention network (LSANet) and other abbreviations should be written like this throughout the paper.

Ans: Thank you for your valuable suggestion. Changes incorporated in the revised manuscript.
2. Also, usage of personal pronoun “We” should also be avoided throughout the paper.

Ans: Thank you for your valuable suggestion. Changes incorporated in the revised manuscript.

3. Authors are suggested to solidify their introduction section further some latest work on the proposed topic are: “A deep recurrent learning-based region-focused feature detection for enhanced target detection in multi-object media”, “Enhancing image categorization with the quantized object recognition model in surveillance systems” and “Improved camouflaged detection in the large-scale images and videos with minimum boundary contrast in detection technique”.

Ans: Thank you for your valuable suggestion. The deep learning techniques for edge and boundary enhancement are added in the revised manuscript.

4. Authors should include a clear problem statement and articulate how LSANet directly addresses an unmet challenge in HSI classification.

Ans: Thank you for your valuable suggestion. The problem statement and how LSANet addresses an unmet challenge in HSI classification have been added in the revised manuscript as follows.

Hyperspectral images contain rich spatial and spectral features that are utilized for several applications, including precise agriculture, cancer diagnosis, surveillance, etc. For the land cover classification, pixels are labelled based on the spatial and spectral characteristics. In addition, it poses a high-dimension and complex spectral-spatial correlation that must be carefully utilized for classification. Traditional CNNs excel at exploring spectral and spatial features but lack long-range dependency on the spatial and spectral features, thus often leading to misclassification of the land covers at boundaries and edge regions. At the same time, transformer-based methods provide global co-relation to the feature map but require a high volume of data for training the model. In addition, the computation costs of the classical ViT attention mechanism are quadratic in time. This study designed LSANet with three major components: 3DCNN, 2DCNN, and limpid shape attention-based ViT encoder. The 3D-CNN is designed using the convolution of size 64, kernel size 3x3, followed by BN (batch normalization) and ReLu activation function. A moderate number of filters, such as 64, ensures diverse features from different filters. At the same time, a small kernel size of 3x3 captures fine-grained spatial features from the edge and boundary region. Moreover, batch normalization stabilizes the training, and ReLu activation allows RSANet to learn the complex patterns of the HSI. The features extracted from the 3D-CNN are reshaped and passed to the 2D-CNN module for spatial feature refinement. The 2D-CNN block contains two convolution layers, one with the convolution of size 128 and the kernel of size 3x3, followed by BN and ReLu activation function and zero padding. The second convolution layer has 256 filters, kernel size 3x3 and ReLu activation function. The feature obtained from the 2D-CNN block is flattened, and tokens are generated for the ViT encoder. The limpid shape attention block captures rich contextual information by splitting it into multiple limpid shape blocks, each of which has equal rows and columns to capture self-attention. In addition, LSANet is GPU friendly and parallel computation of attention score can be performed, which results in less training time.

5. Improve resolution and labeling of all figures.

Ans: Thank you for your valuable suggestion. Image resolution and labelling have been improved in the revised manuscript.
6. Add 2–3 sentences after each ablation result table to interpret key findings. Explain why each component contributes to performance improvement.

Ans: Thank you for your valuable suggestion. After each ablation study table key findings have been added in the revised manuscript.

Before Table 11 following key findings have been added.

The SA mechanism in the ViT calculates the attention score across all pair tokens and can provide global attention. However, it may miss the local mid-level features essential for HSI classification. Moreover, ASA calculates attention in rows and columns at a time. Due to this, the diagonal features may be missing. At the same time, CSA has a cross pattern that is broader along the axes but narrower in terms of directional coverage. Moreover, the LSA calculates attention in all directions and is more adaptive to provide a long-range dependency on spatial features.

Before Table 12 following key findings have been added.

The fixed positional encoding (FPE) technique, such as sinusoidal, uses a sine or cosine function to generate the encoding in the image at different frequencies. Another fixed positional encoding technique, learned positional encoding (LPE), provides a model to learn positional encoding from the positional encoding vector during training. However, after training, it remains fixed. Our CPE generates dynamic positional encoding using depth-wise convolution from the input feature map,which makes them effiient and flexible across different resolution

7. Justify why zero-padding was chosen over alternative techniques (e.g., reflection padding or learned embeddings) and its impact on edge classification.

Ans: Thank you for your concern. Let the HSI image, which has height P, width Q and spectral bands D. Principal component analysis (PCA) is applied to reduce the dimension of the HSI to. After that, patches of dimension $n\times n\times b$ is extracted. Each patch is formed around a pixel, which serves as the patch's focal point. Furthermore, a padding procedure is applied to generate a spatial foundation for these pixels. The extracted patch of size 9x9 is passed to the 3DCNN block for the spectral and spatial features. After that, feature map is reshaped and passed to the 2DCNN block for the enhancement of the spatial features. The first 2DCNN layer contains zero padding to preserve the spatial resolution of the pixel-wise HSI classification. At the same time, it does not increase the computation burden since only zeros are added around the feature map. Moreover, it ensures that the output dimension matches the input. The reflection padding creates mirrors of the edge and boundary region. However, in the HSI, the pixel pattern of land covers differs, and performing reflection padding may overlap the different class pixels. At the same time, set parameters are used to represent paddings in the learned embedding training. Thus, it will increase the training time and computation burden. The zero padding does not contain meaningful information on the edge and boundary region, which may reduce the classification performance. ViT encoders have been utilized to mitigate the problem and provide a global correlation to the overlapping patches.
